# Research on land cover classification of multi-source remote sensing data based on improved U-net network

**DOI:** 10.1038/s41598-023-43317-1

**Published:** 2023-09-28

**Authors:** Guanjin Zhang, Siti Nur Aliaa binti Roslan, Ci Wang, Ling Quan

**Affiliations:** 1 Department of Civil Engineering, Faculty of Engineering, University Putra Malaysia, 43400 Serdang, Selangor Malaysia; 2 College of Resource and Environment, Anhui Science and Technology University, Chuzhou, 233100 China; 3 School of Physics and Electronic-Electrical Engineering, Ningxia University, Yinchuan, 750021 China

**Keywords:** Ecology, Environmental sciences

## Abstract

In recent years, remote sensing images of various types have found widespread applications in resource exploration, environmental protection, and land cover classification. However, relying solely on a single optical or synthetic aperture radar (SAR) image as the data source for land cover classification studies may not suffice to achieve the desired accuracy in ground information monitoring. One widely employed neural network for remote sensing image land cover classification is the U-Net network, which is a classical semantic segmentation network. Nonetheless, the U-Net network has limitations such as poor classification accuracy, misclassification and omission of small-area terrains, and a large number of network parameters. To address these challenges, this research paper proposes an improved approach that combines both optical and SAR images in bands for land cover classification and enhances the U-Net network. The approach incorporates several modifications to the network architecture. Firstly, the encoder-decoder framework serves as the backbone terrain-extraction network. Additionally, a convolutional block attention mechanism is introduced in the terrain extraction stage. Instead of pooling layers, convolutions with a step size of 2 are utilized, and the Leaky ReLU function is employed as the network's activation function. This design offers several advantages: it enhances the network's ability to capture terrain characteristics from both spatial and channel dimensions, resolves the loss of terrain map information while reducing network parameters, and ensures non-zero gradients during the training process. The effectiveness of the proposed method is evaluated through land cover classification experiments conducted on optical, SAR, and combined optical and SAR datasets. The results demonstrate that our method achieves classification accuracies of 0.8905, 0.8609, and 0.908 on the three datasets, respectively, with corresponding mIoU values of 0.8104, 0.7804, and 0.8667. Compared to the traditional U-Net network, our method exhibits improvements in both classification accuracy and mIoU to a certain extent.

## Introduction

The utilization of remote sensing images with wide coverage and fast imaging has become increasingly common due to advancements in remote sensing technology^1^, the most representative ones are undoubtedly optical remote sensing images and SAR remote sensing images. Optical remote sensing images offer rich spectral information and relatively high resolution^2,3^, these features play a very important role in the differentiation of similarly colored terrains^4^. Similarly, SAR remote sensing images also contain rich feature backscattering characteristics, and the SAR sensor has strong penetration, image acquisition is not affected by the weather^5^, and enables all-day, all-weather observation of the Earth's surface. Therefore, two different remote sensing images are widely used in land cover classification studies. In the beginning, many machine learning algorithms were used in feature classification studies. For example, Heumann B W et al.^6^ achieved a classification accuracy of 94% on WorldView-2 data using an OBIA classification method combining decision trees and support vector machines (SVM)^7^. Zhao et al.^8^ used Sentinel-2 imagery as experimental data to accurately classify land cover in the Red River Delta region of Vietnam using three methods, namely Random Forest (RF)^9^, K-means^10^ and SVM, all of which have classification accuracies of more than 90%. Similarly, Zhao et al.^11^ achieved a classification accuracy of 88.97% on dual-polarized RISAT-1 SAR data using SVM. Although machine learning-based classification methods are simple and fast, the generalization ability of the network cannot meet people's requirements and it is difficult to complete the end-to-end semantic segmentation task^12^.

The rapid development of artificial intelligence has made deep learning a cutting-edge technique in feature classification research. Many studies have shown that, compared with traditional machine learning, the unique multi-layer structure of neural networks can extract multi-scale and deep-level feature information, so it can obtain better classification accuracy and effect^13–15^. Therefore, more and more scholars apply deep learning to land cover classification research. For instance, Zhou et al.^16^ introduced the D-LinkNet, a semantic segmentation neural network that expands the sensory field through the addition of inflated convolutions in the center of LinkNet, thereby improving extraction accuracy for road terrains. Aiming at the problem of low classification accuracy due to the small footprint and irregular shape of mines, Zhou et al.^17^ proposed a network model named EG-UNet, which uses an information extraction module to extract tiny objects during the training process and enhances the feature weight information of the edges of the mines by a feature enhancement module, which effectively improves the accuracy of classification. Similarly, Yan et al.^18^ enhanced the DeepLabV3 network by introducing self-attention mechanisms, terrain pyramid structures, and Res Net structures to improve global-scale regional terrain connections and classification accuracy. Zhao et al.^19^ proposed the PSP-Net network, which added a terrain pyramid module to the FCN^20^ network and aggregated contextual information from different regions to enhance global information accessibility. Ma et al.^21^ proposed a Feature Enhancement Network (FENet) for enhancing buildings and water bodies based on the self-attention mechanism, and the accuracy of this network is higher than existing models. Li et al.^22^ introduced asymmetric convolutions to the U-Net network to enhance the terrain representation and extraction capability of the convolutional layer, effectively addressing terrain extraction rate and under-utilization issues. Hu et al.^23^ proposed MCSGNet by introducing an information guidance module and a feature fusion module on the encoder-decoder architecture, which effectively solved the problems of feature information loss and weak model generalization.

In addition to that, Fu et al.^24^ enhanced the DeepLabV3 + network for high-resolution remote sensing images by incorporating the MobileNetV2 network as the backbone terrain extraction network, introducing attention mechanisms and focus loss balancing. Lv et al.^25^ addressed the detection challenges in non-uniform remote sensing images by incorporating a multi-scale convolution module and focus dice combination loss function into the U-Net network framework, significantly improving detection accuracy. Aiming at the problems of large number of parameters and insufficient attention to the focus region in U-Net++ network, Niu et al.^26^ introduced the attention mechanism on the basis of U-Net++ by removing the depth supervision and replacing the convolutional block with RegNet, which effectively reduces the number of parameters in the network and solves the problem of insufficient attention to the focus region. Alicia Passah et al.^27^ propose a novel, simple network model that outperforms existing models in terms of both parameter complexity and classification accuracy by drawing on the properties of the InceptionV3 and MobileNet models and combining deep and unit-separable convolution.

While both traditional and deep learning methods have made significant progress in land cover classification, most research data have relied solely on visible and SAR data. Several studies have highlighted the insufficiency of using a single data source for achieving reliable classification accuracy. Moreover, traditional neural networks for land cover classification face challenges such as a large number of network parameters, insufficient focus on key regions, and difficulties in capturing global dependencies. In this study, our approach involves enhancing the land cover classification process by combining optical and SAR images using wavebands. We propose an encoder-decoder framework as the terrain extraction network, utilizing Leaky ReLU as the activation function. Additionally, we incorporate an attention mechanism into the network structure and employ convolutional replacement pooling to reduce the number of parameters in the network. To evaluate the effectiveness of our approach, we compare its classification accuracy with that of the U-Net on separate optical and SAR datasets, as well as a combined dataset comprising both bands. The results demonstrate that our improved network not only reduces the number of network parameters but also improves classification accuracy.

## Study area and data pre-processing

### Study area

**Figure 1:**
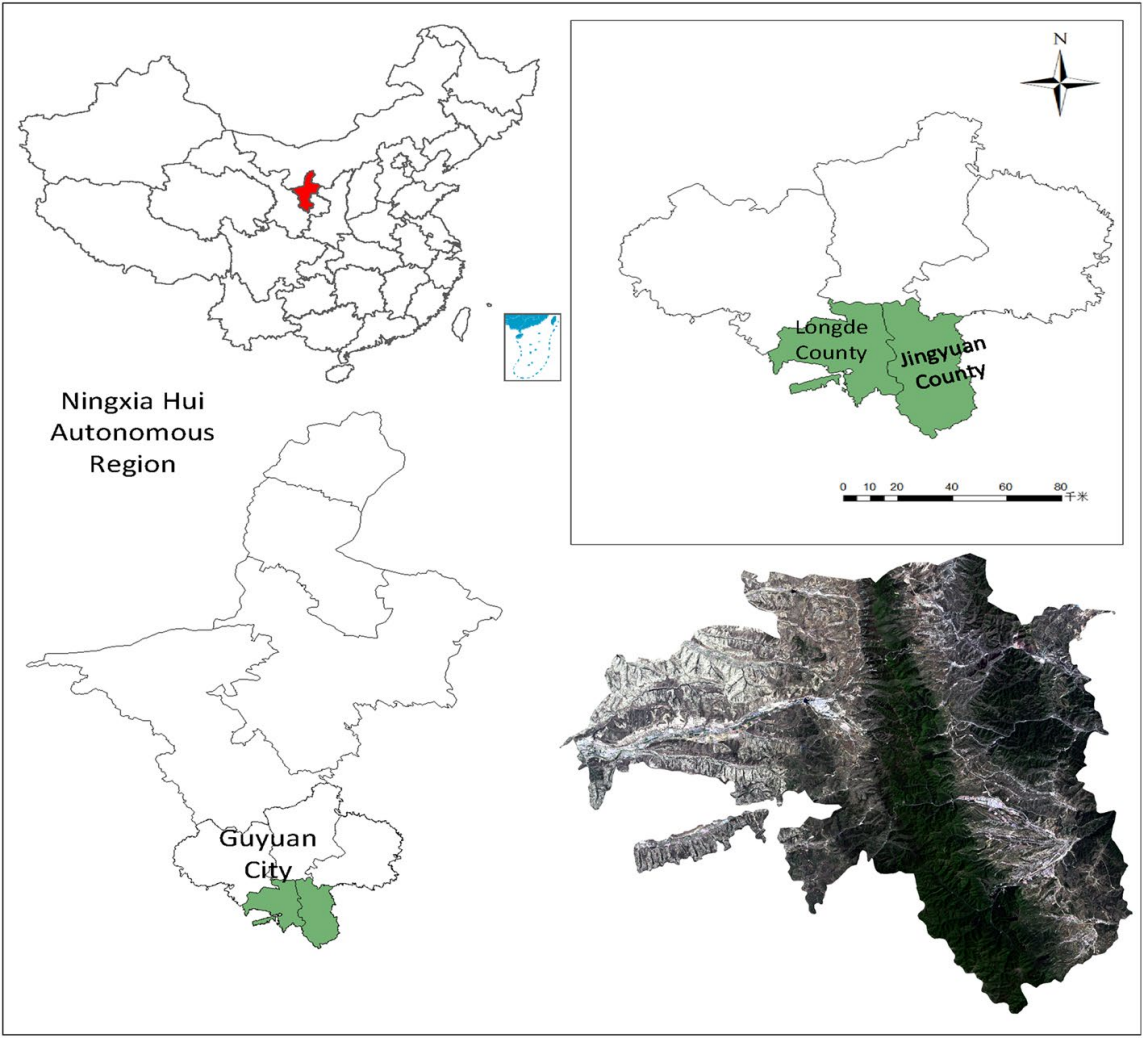
Schematic diagram of the geographical location of the study area.

The study area for this research encompasses Jingyuan and Longde counties, situated in Guyuan City, Ningxia Hui Autonomous Region. The geographical locations of these counties are depicted in Fig. 1. Jingyuan County is positioned at the eastern foothills of Liupan Mountain, spanning from longitude 106° 12′ ~ 106° 29′ E and latitude 35° 15′ ~ 35° 38′N. On the other hand, Longde County is situated at the western foothills of Liupan Mountain, ranging from longitude 105° 48′ ~ 106° 15′ E and latitude 35° 21′ ~ 35° 47′ N. The terrain exhibits higher elevation in the east and lower elevation in the west. The region experiences a transitional climate between semi-humid and semi-arid conditions in the middle temperate monsoon zone. Known for its climatic characteristics, the area is described as having penetrating winds during muggy summers and a lack of blooming snow in fragrant springs. Jingyuan and Longde counties are strategically located at the center of Shanxi, Gansu, and Ningxia provincial capitals, facilitating convenient transportation and boasting developed tourism. Therefore, the present study selects Jingyuan and Longde counties as the focal area to conduct land cover classification, given its significance for the economic development and ecological civilization construction of both counties and the entire region.

### Remote sensing data and pre-processing

For this study, the chosen SAR remote sensing data consists of Sentinel-1A intensity data acquired in August 2020, using the IW imaging mode. The Sentinel-1 satellite is equipped with a C-band synthetic aperture radar (SAR) that offers four imaging modes, enabling all-weather and all-day image acquisition of the Earth's surface. On the other hand, the selected optical remote sensing data comprises Level-2A Sentinel-2A data acquired in November 2020, with cloud coverage of less than 5%. The Sentinel-2 satellite provides three ground resolutions (10 m, 20 m, and 60 m) and covers 13 spectral bands of information.

The pre-processing of the Sentinel-1 images involves several steps using SARscape, including multi-view processing, filtering, geocoding, radiometric calibration, and cropping of the study area. Since the average elevation of the study area ranges from 1608 to 2942 m, DEM data specific to the area is incorporated during geocoding and radiometric calibration to eliminate topographic distortion. It is worth noting that ENVI 5.3 does not support the Sentinel-2A data format. Therefore, before processing with ENVI, the data is initially resampled using SNAP and then converted to the ENVI format to generate RGB three-channel images through band synthesis. The synthesized RGB image has a resolution of 10 m, while the resolution of the Sentinel-1 SAR data is 20 m. To ensure compatibility, the projection coordinates of both datasets are converted to the same system (UTM-ZONE-48N under the WGS-84 ellipsoid) after completing the data preprocessing operations. Subsequently, the Sentinel-1 SAR data is resampled to a 10 m resolution, and finally, the two datasets are merged using ENVI classic.

### Dataset production

In addition to vegetation, the study area exhibits non-vegetated areas due to human activities and the natural environment. These non-vegetated areas primarily include buildings, water bodies, roads, and cultivated land. Accordingly, this research paper classifies the terrain types in the study area into eight categories. Table 1 presents the terrain types and corresponding label information.

**Table 1:** Terrain type and label information table.

Terrain type	Tags	Label corresponding arrays	Label color
Building	Building	(0, 128, 0)	
Water	Water	(128, 128, 0)	
Bare area	ba	(128, 0, 128)	
Crop	Crop	(64,0,0)	
Road	Road	(128, 0, 0)	
Coniferous forest	cf	(0, 0, 128)	
Broad-leaved forest	blf	(0, 128, 128)	
Theropencedrymion	th	(128,128,128)	

The obtained study area images, which were cropped based on the SHP file, exhibit irregular shapes. These images are further cropped into 256 × 256-sized images. Images with gaps are discarded, and different areas are assigned corresponding labels using Label me software. Due to the geographic characteristics of the study area, terrains such as water bodies and roads are less represented and scattered compared to terrains like cultivated land, buildings, and deciduous forests. Additionally, the three self-made datasets in this research are relatively small in scale, which may lead to overfitting if directly used for training deep learning networks. To address these issues and increase the dataset size, as well as achieve a more balanced distribution of object types, the Sentinel-2 optical dataset, the Sentinel-1 SAR dataset, and the band combination dataset are enhanced through a 90° rotation transformation, mirror flipping, and adjustments to contrast and color enhancement in the images, respectively. After a series of data enhancement operations, the number of images in all three datasets reached 2670, and these images were used to randomly divide the datasets into training, validation and test sets in the ratio of 7:2:1. This completes the construction of the dataset for remote sensing image feature classification research.

## Improved U-net network model

**Figure 2:**
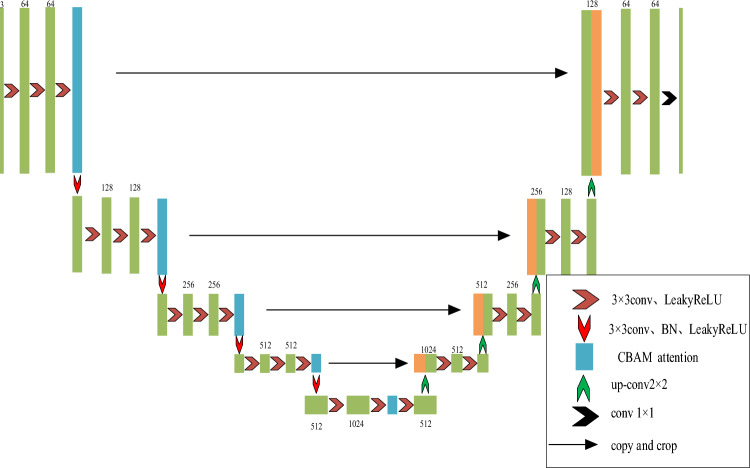
Improved structure diagram of U-Net.

To enhance the classification accuracy and training efficiency of the traditional U-Net network, several improvements are made in this study. Firstly, instead of using pooling layers for down-sampling, a convolution operation with a step size of 2 is employed. This modification aims to address the issue of partial information loss and potential gradient death during the training process. Secondly, the U-Net network's terrain extraction stage incorporates the CBAM (Convolutional Block Attention Module (CBAM)) attention mechanism. This addition enhances the network's focus on terrain information of the objects. Lastly, we use the Leaky ReLU function instead of the ReLU function in the original U-Net network as the activation function for the improved network. This step helps mitigate the impact of gradient death on classification accuracy during network training. Figure 2 illustrates the enhanced structure of the U-Net network.

### Optimized down-sampling module

Convolutional and pooling layers are integral components of neural networks. Convolution is responsible for terrain extraction by multiplying the terrain map with corresponding positions of the convolution kernel and summing the results. Notably, the terrain map size remains unchanged after convolution, but the number of channels increases. On the other hand, the pooling layer is typically positioned between convolutional layers and reduces the terrain map size while retaining the same number of channels. Convolution effectively compresses the terrain map size through techniques like step size and padding, achieving the function of size compression without the need for pooling.

Following the introduction of ResNet networks, the replacement of pooling layers with convolutional layers of step size 2 gained popularity. In 2015, Jost Tobias Springenberg et al.^28^ proposed ACNet, a network that eliminates pooling layers and gradually reduces the terrain map size using multiple convolutional layers. Experimental results demonstrate that ACNet achieves comparable performance to traditional convolutional neural networks while avoiding information loss and overfitting issues associated with pooling layers. In 2017, Szegedy et al.^29^ discovered that employing convolution instead of pooling reduces the total number of parameters in ResNet, leads to faster convergence, and yields better performance. Similarly, in 2019, Marjan et al.^30^ presented a novel architecture based solely on 2D convolutional neural networks, which excludes pooling layers. Experimental results showcased the high performance of the 2D-CNN networks in the absence of pooling layers. The structure of the improved network is illustrated in Table 2, while Table 3 provides information on the number of network parameters and floating point computations before and after the improvements.

**Table 2:** Structure table of the improved network.

Steps	Operations	Output size
Input	No-operation	256 × 256 × 3
Step 1	2*(Conv BN Leaky ReLU) + CBAM	256 × 256 × 64
Step 2	2*(Conv BN Leaky ReLU) + CBAM	128 × 128 × 128
Step 3	2*(Conv BN Leaky ReLU) + CBAM	64 × 64 × 256
Step 4	2*(Conv BN Leaky ReLU) + CBAM	32 × 32 × 512
Step 5	2*(Conv BN Leaky ReLU) + CBAM	16 × 16 × 512
Step 6	upconv	32 × 32 × 512
Step 7	2*(Conv BN Leaky ReLU) + up conv	64 × 64 × 256
Step 8	2*(Conv BN Leaky ReLU) + up conv	128 × 128 × 128
Step 9	2*(Conv BN Leaky ReLU) + up conv	256 × 256 × 64
Step 10	2*(Conv BN Leaky ReLU) + conv + Sigmoid	256 × 256 × 2

**Table 3:** Computation of the number of parameters and floating points of the network before and after improvement.

Models	Total params	GFLOPs
U-Net	13,395,849.0	31,152,668,672.0
Ours	12,422,816.0	25,540,461,728.0

As can be seen in Table 3, the number of parameters and floating-point computation of the network is reduced by using convolution instead of pooling and by changing the number and size of the original convolution kernel structure. Specifically, when compared to U-Net, the parameter quantity of improved network is reduced by 7.26%, and the floating-point computation is reduced by 18.02%.

### Add CBAM attention mechanism

**Figure 3:**
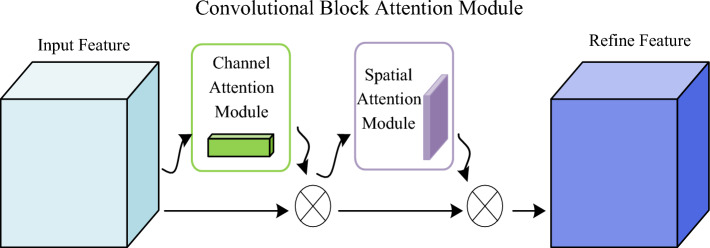
Structure diagram of CBAM.

The attention mechanism refers to the process of shifting focus from less significant parts to the most important parts. Within the realm of deep learning, the attention mechanism can be viewed as a dynamic selection process that assigns varying weights to input data based on its importance. This adaptive weight assignment enhances attention toward crucial components. The attention mechanism has found wide application in various computer vision tasks, and recent studies in neural networks have demonstrated its effectiveness in improving network performance^31^. To further enhance attention toward terrain information of small area terrains, this study incorporates a convolutional block attention mechanism during the terrain extraction stage. As proposed by Woo et al.^32^, this attention mechanism combines both channel attention and spatial attention. By computing attentional terrain maps in both channel and spatial dimensions from the given terrain maps, these maps are then multiplied with the input terrain maps to assign adaptive weights. The calculation formula is depicted in Eqs. (1) and (2), while Fig. 3 illustrates the structure of this attention mechanism.

1 $$ F^{\prime} = M_{{\text{c}}} (F) \otimes F $$

2 $$ F^{\prime\prime} = M_{s} (F^{\prime}) \otimes F^{\prime} $$

where $\otimes$ denotes the element-wise multiplication operation, whereby the values of channel attention and spatial attention are multiplied together in their respective dimensions. The resulting product is denoted as $F^{\prime\prime}$ and represents the final output value.

The convolutional block attention mechanism consists of two components: the channel attention mechanism^33^ and the spatial attention mechanism^34^. The channel attention mechanism plays a vital role in automatically learning terrain weights. It enhances the network's ability to extract useful information from specific channels while suppressing the extraction ability for channels with lesser significance. This helps reduce the interference caused by irrelevant information and improves the overall accuracy rate. The formula for the channel attention mechanism is presented in Eq. (3).

3 $$  \begin{aligned}   M_{c} (F) &  = \sigma (MLP(AvgPool(F)) + MLP(MaxPool(F))) \\    {\kern 1pt} {\kern 1pt} {\kern 1pt}  &  = \sigma (W_{1} (W_{0} (F_{{avg}}^{c} )) + W_{1} (W_{0} (F_{{\max }}^{c} ))) \\  \end{aligned}    $$

where $\upsigma$ denotes the activation function Sigmoid, $AvgPool$ denotes the global average pooling operation, $MaxPool$ denotes the global maximum pooling operation, $W_{1}$ and $W_{2}$ denotes the $MLP$ weights shared by both.

The spatial attention module (SAM) serves as a complement to the channel attention mechanism. It compresses the channels, conducts global average pooling and maximum pooling operations, and learns a weight matrix from the terrain map with dimensions H × W in the two-dimensional plane. Subsequently, the weight matrix is concatenated with the original terrain map. This process enhances the attention toward valuable terrains while attenuating or disregarding irrelevant terrains. The equation representing the spatial attention mechanism is presented as Eq. (4).

4 $$ \begin{aligned}   M_{s} (F) &  = \sigma (f^{{7*7}} ([AvgPool(F);MaxPool(F)]) \\    {\kern 1pt}  &  = \sigma (f^{{7*7}} ([F_{{avg}}^{{\text{s}}} ;F_{{\max }}^{{\text{s}}} ])) \\  \end{aligned}   $$

where $\upsigma$ denotes the activation function Sigmoid and $f^{7*7}$ denotes the convolution operation with a convolution kernel size of 7 × 7.

### Optimizing the activation function

**Figure 4:**
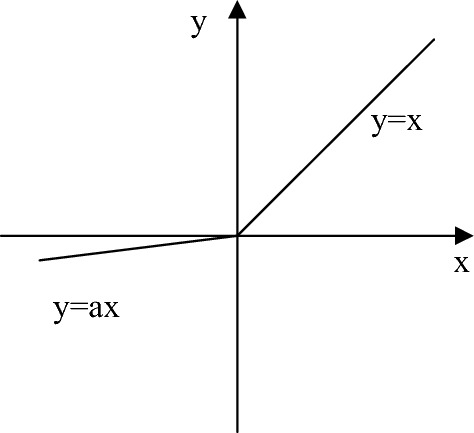
The Leaky ReLU function.

In a deep learning model, the neural network weights are trained using loss backpropagation^35^. However, due to the presence of large areas in the acquired images and varying percentages of different terrain types, the training process requires more rounds to address the issue of uneven percentages. However, the activation function in the original U-Net network is the ReLU function, and when $x < 0$, the network has the problem of gradient vanishing parameters not updating, which will affect the accuracy of classification. To address the issue of gradient death during network training, this study adopts the Leaky ReLU^36^ function as the activation function. Unlike the ReLU function, the Leaky ReLU function encompasses the entire real number domain and introduces a small linear component in the negative semi-axis, overcoming the problem of weight parameter stagnation encountered when using the ReLU function. Equation (5) illustrates the formula for the Leaky ReLU function, and Fig. 4 depicts its function graph.

5 $$ Leaky\,{\text{Re}} LU(x) = \left\{ \begin{gathered} {\text{a}}x{\kern 1pt} {\kern 1pt} {\kern 1pt} {\kern 1pt} {\kern 1pt} {\kern 1pt} {\kern 1pt} {\kern 1pt} {\kern 1pt} {\kern 1pt} {\kern 1pt} x \le 0 \hfill \\ x{\kern 1pt} {\kern 1pt} {\kern 1pt} {\kern 1pt} {\kern 1pt} {\kern 1pt} {\kern 1pt} {\kern 1pt} {\kern 1pt} {\kern 1pt} {\kern 1pt} {\kern 1pt} {\kern 1pt} {\kern 1pt} {\kern 1pt} {\kern 1pt} x{ > 0} \hfill \\ \end{gathered} \right. $$

## Experimental results and analysis

### Evaluation index and experimental environment

#### Evaluation index

In remote sensing image land cover classification tasks, evaluation metrics such as Pixel Accuracy (PA), mean Pixel Accuracy (mPA), Recall, and mean Intersection over Union (mIoU) are commonly employed to quantitatively assess the performance of the network. In this study, we select PA and mIoU as the evaluation indices for three distinct types of remote sensing datasets utilized in land cover classification tasks. The formulas for PA and mIoU are provided in Eqs. (6) and (7).

6 $$ PA = \frac{TP + TN}{{TP + FP + FN + TN}} $$

7 $$ mIoU = \frac{1}{N}\sum\nolimits_{i = 1}^{N} {IoU}_{i} $$

In Eq. (6), $TP$ indicates that the actual positive samples are classified as positive samples, $TN$ indicates that the actual negative samples are classified as negative samples, $FP$ indicates that the actual negative samples are classified as positive samples, $FN$ indicates that the actual positive samples are classified as negative samples. A higher value of PA indicates greater proximity between the predicted and true values, indicating better performance of the network.

In Eq. (7), $N$ denotes the number of categories classified, and $IOU_{i}$ denotes the IOU of the *i*st classified category. mIoU larger values indicate the higher accuracy of the network classification.

#### Experimental environment

The experimental setup in this study consists of both hardware and software environments. The hardware environment utilized a 64-bit Windows system with an Intel i7-12700 CPU, an NVIDIA GeForce RTX3060Ti GPU, and 8 GB of video memory. The software environment is based on PyTorch, a deep learning framework, along with its corresponding Python library.

Specific parameters were set for the experiments. The initial learning rate was assigned as 0.0001, weight decay was set to 1e-5, and a natural exponential decay polynomial was employed for the learning rate decay strategy. The input image size was fixed at 256 × 256 pixels, with a batch size of 4. The U-Net network underwent 150 iterations, while the improved network underwent 200 iterations. The Adam algorithm was chosen as the network parameter update algorithm.

### Classification results and analysis based on multi-source remote sensing data

#### Classification results and analysis of Sentinel-2 dataset

**Figure 5:**
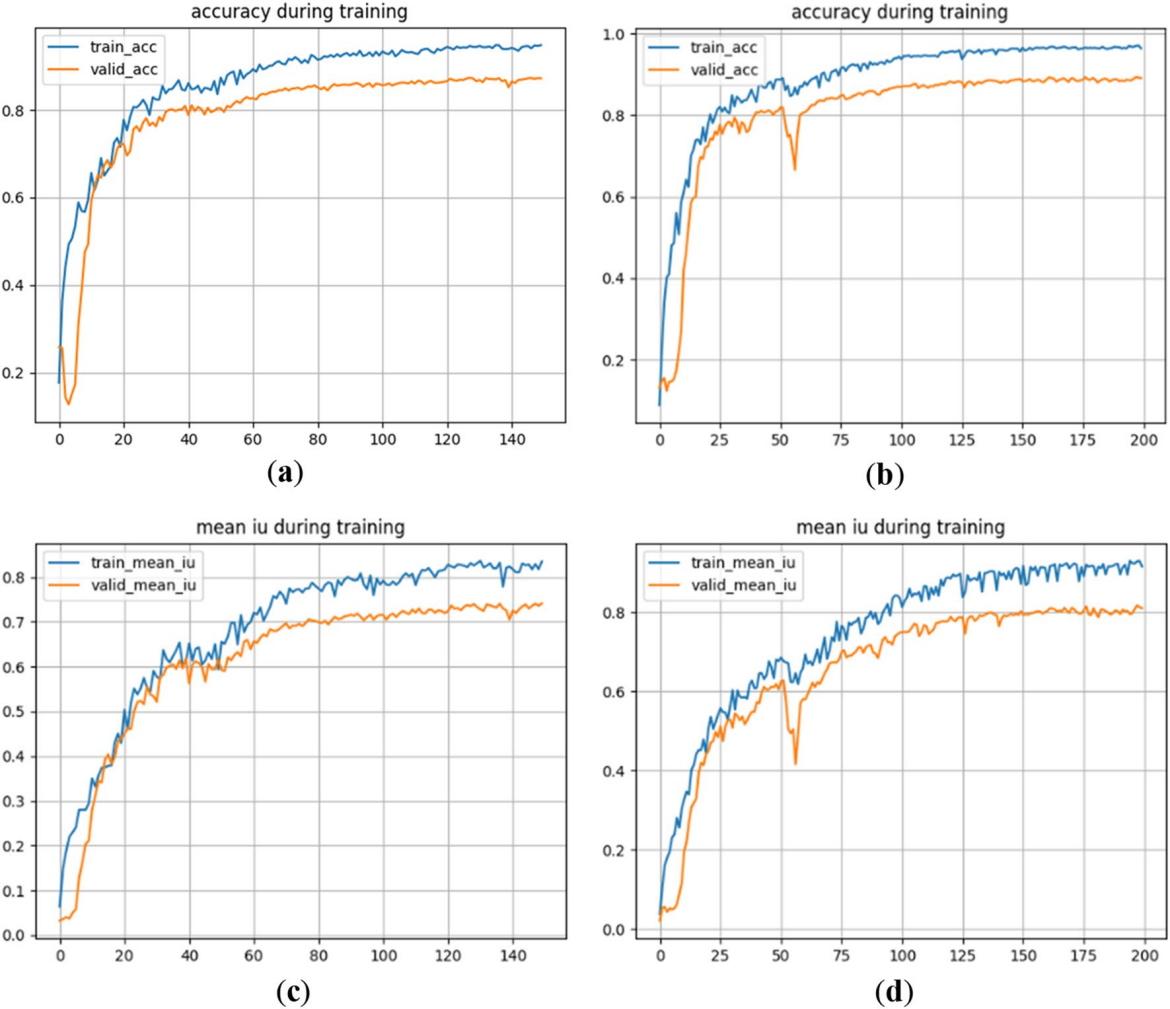
Comparison of two curves of the network before and after improvement under Sentinel-2 dataset. (**a**) Accuracy curve of U-Net network; (**b**) accuracy curve of the improved network; (**c**) mIoU curve of U-Net network; (**d**) mIoU curve of improved network.

The accuracy curves and the average cross-merge ratio curves of U-Net and the improved network under the Sentinel-2 dataset are shown in Fig. 5.

Figure 5 demonstrates that while the accuracy curve and average cross-comparison ratio of the improved network display more fluctuations during the 55th to 60th iterations, the overall fluctuation is lower before convergence. Moreover, the accuracy curve of the network gradually levels off and converges after 125 iterations. Both the training and validation sets exhibit crossover phenomena in their accuracy and average cross-ratio curves. Once both the U-Net network and the improved network converge, the corresponding values for pixel accuracy and average cross-comparison ratio are presented in Table 4.

**Table 4:** Values of accuracy and average cross-merge ratio of the network before and after improvement.

Models	Training set		Validation set	
	Accuracy	mIoU	Accuracy	mIoU
U-Net	0.9477	0.8348	0.8721	0.7411
Ours	0.9635	0.9159	0.8905	0.8104

Based on the information provided in Table 4, it can be observed that the improved network achieves higher training and validation accuracies compared to the U-Net network with the Sentinel-2 dataset. Specifically, the training accuracy and validation accuracy of the improved network, respectively, 1.35% and 2.41% higher than those of the U-Net. Furthermore, the average cross-merge ratio for training and validation is respectively 5.14% and 3.08% higher in the improved network compared to the U-Net.

**Figure 6:**
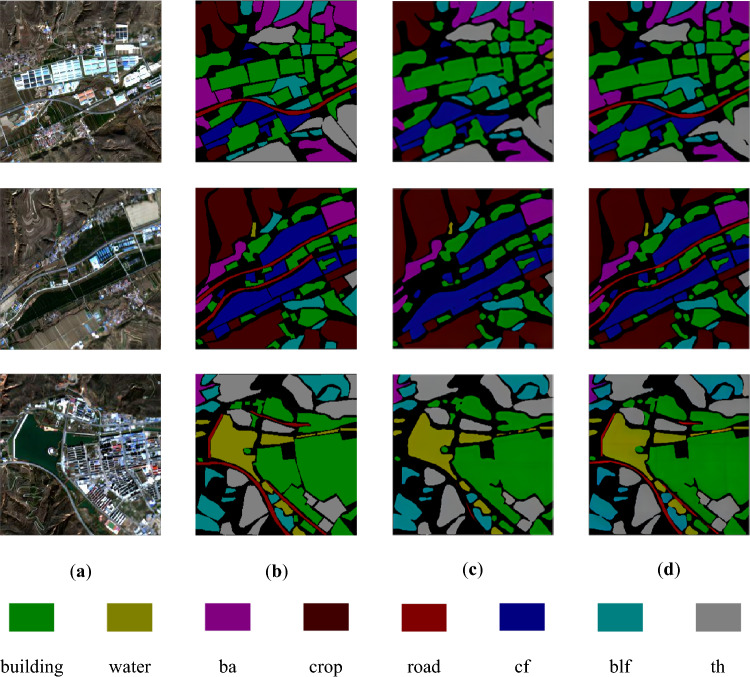
Comparison of network prediction results before and after improvement. (**a**) Original image; (**b**) real labels; (**c**) results of U-Net; (**d**) results of improved network.

A comparison of the prediction results of the U-Net network and the improved network is shown in Fig. 6.

Upon observing (c) and (d) in Fig. 5, it is evident that the U-Net network performs well in classifying the seven terrain types, except for roads. Although there is a slight discrepancy between the recognized range of the seven terrains and their actual labels, the overall recognition effect is satisfactory. However, the U-Net network encounters difficulties in recognizing roads, resulting in missing scores. On the other hand, the improved network demonstrates proficiency in classifying not only the seven terrain types but also roads accurately. Furthermore, the improved network exhibits a narrower gap between the recognized range of the eight terrains and their real labels.

#### Classification results and analysis of sentinel-1 dataset

**Figure 7:**
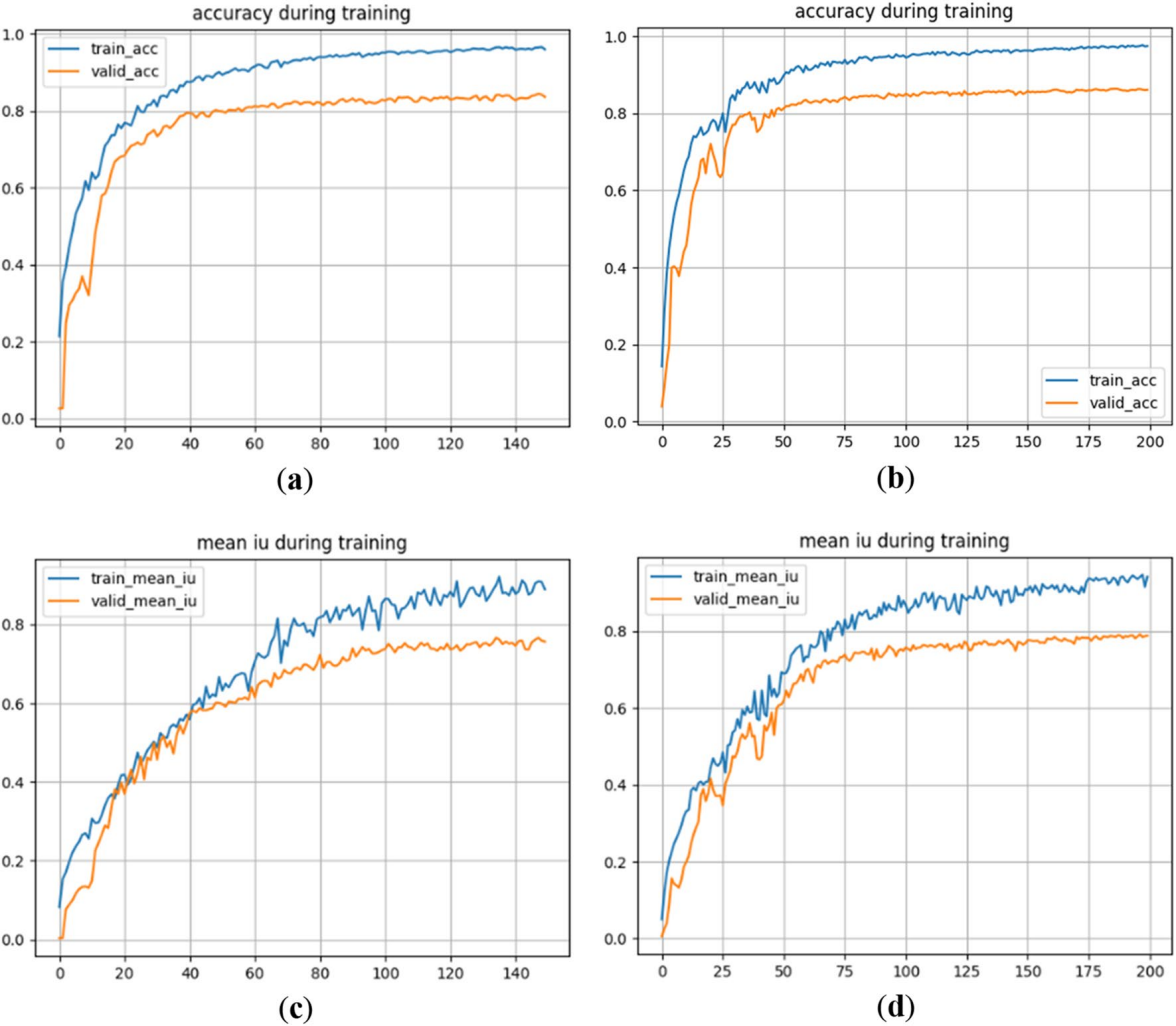
Comparison of two curves of the network before and after improvement under Sentinel-2 dataset. (**a**) Accuracy curve of U-Net network; (**b**) Accuracy curve of the improved network; (**c**) mIoU curve of U-Net network; (**d**) mIoU curve of improved network.

The accuracy curves and the average cross-merge ratio curves of U-Net and the improved network under the Sentinel-2 dataset are shown in Fig. 7.

By examining Fig. 7, it is apparent that the improved network exhibits greater fluctuations in the accuracy curve and average cross-merge ratio during the training process. The accuracy curve of the improved network tends to level off and converge after 175 iterations. In comparison, although the accuracy curve of the U-Net network converges earlier, there exists a wider gap between the accuracy curves of the training set and the validation set. Moreover, the average cross-merge ratio curve of the U-Net network displays more frequent fluctuations and larger amplitudes, with occurrences of crossover phenomena. Once both the U-Net network and the improved network reach convergence, the pixel accuracy and average cross-merge ratio values for both networks are provided in Table 5.

**Table 5:** Values of accuracy and average cross-merge ratio of the network before and after improvement.

Models	Training set		Validation set	
	Accuracy	mIoU	Accuracy	mIoU
U-Net	0.9606	0.8894	0.8368	0.7566
Ours	0.9741	0.9408	0.8609	0.7874

It can be deduced from the data in Table 5, the training accuracy and validation accuracy of the improved network are 1.35% and 2.41% higher than those of U-Net under the Sentinel-1 dataset, and the average cross-merge ratio for training and validation sets are 5.14% and 3.08% higher than those of U-Net.

**Figure 8:**
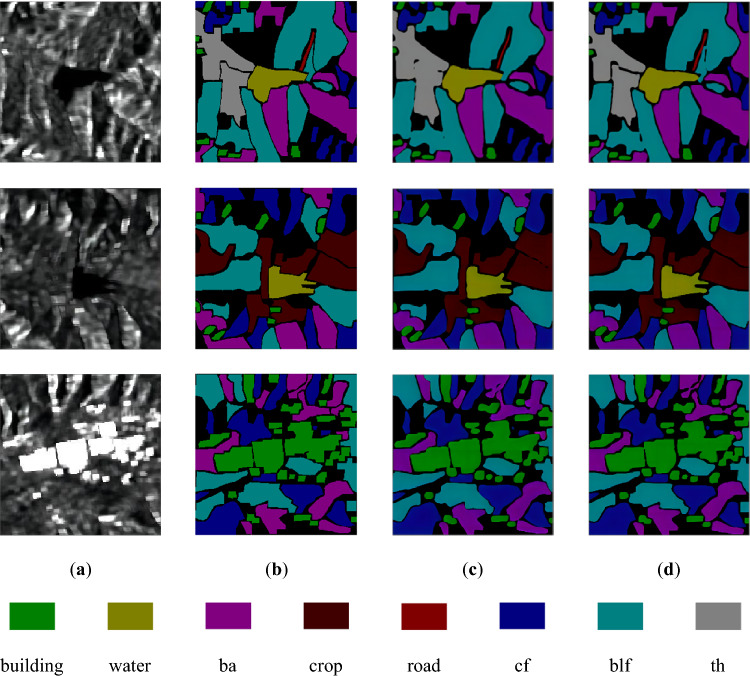
Comparison of network prediction results before and after improvement. (**a**) Original image; (**b**) Real Labels; (**c**) Results of U-Net; (**d**) Results of improved network.

A comparison of the prediction results of the U-Net network and the improved network are shown in Fig. 8.

Upon examining (c) and (d) in Fig. 8, it is evident that the U-Net network performs well in classifying all the terrains in the dataset. Although there is a slight difference between the recognized range of the terrains and their true labels, the overall recognition effect is commendable. Unlike the Sentinel-2 dataset, the U-Net network does not exhibit the issue of missing scores in the Sentinel-1 dataset. Furthermore, when compared to the U-Net network, our proposed network demonstrates smaller errors between the predicted results and the true labels.

#### Classification results and analysis of the combined dataset

**Figure 9:**
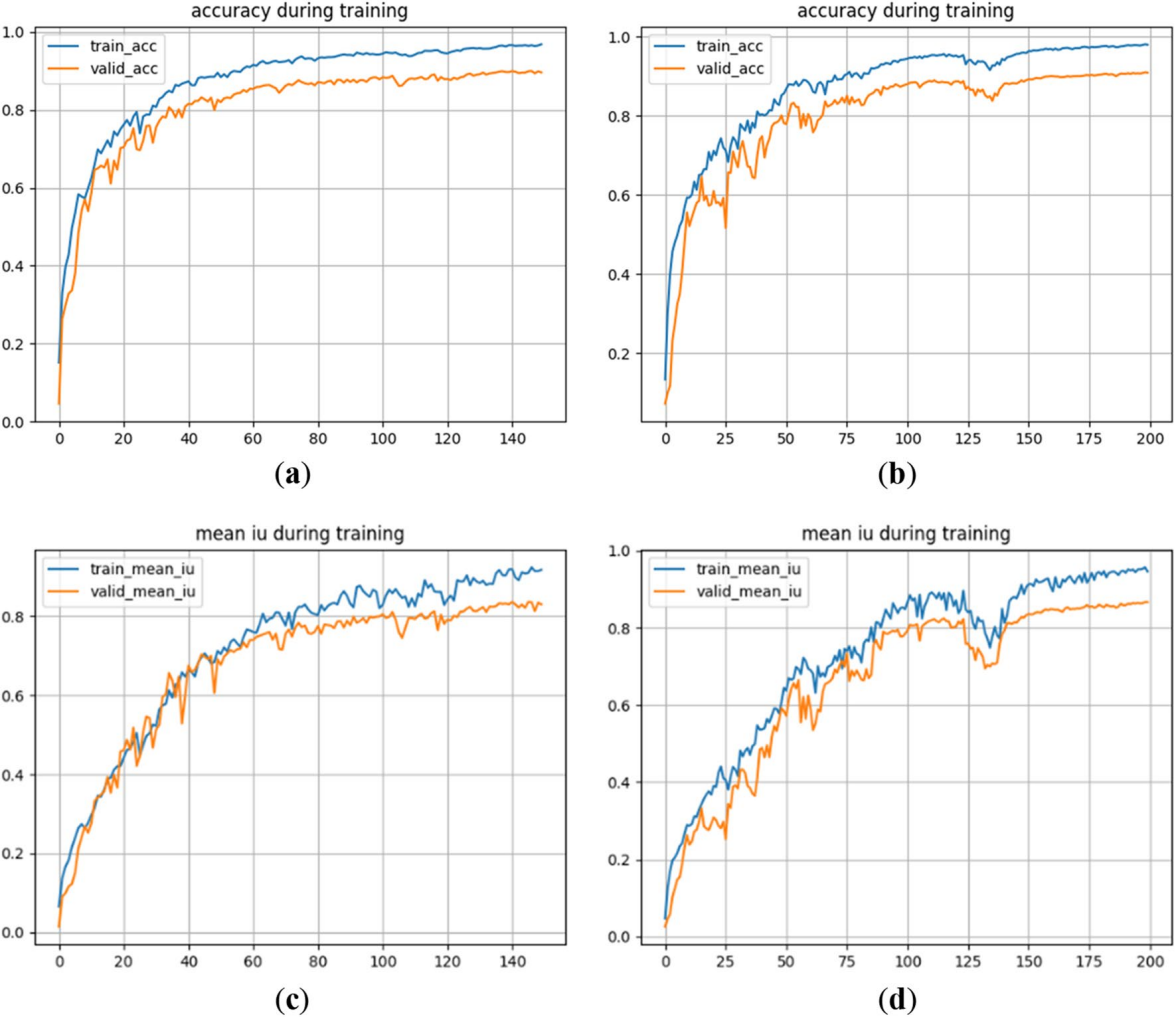
Comparison of two curves of the network before and after improvement under the combined dataset. (**a**) Accuracy curve of U-Net network. (**b**) Accuracy curve of the improved network; (**c**) mIoU curve of U-Net network; (**d**) mIoU curve of improved network.

The accuracy curves and the average cross-merge ratio curves of U-Net and the improved network under the combined dataset are shown in Fig. 9.

Upon examining Fig. 9, it is evident that the accuracy curves of both networks exhibit a more rapid rise at the initial stages of the training process. Additionally, the average cross-comparison curves of the two networks display more fluctuations. After 163 iterations, the accuracy curves of our proposed network start to level off and converge. Although the convergence speed is slower and the training time is longer for our network, we do not encounter the same issue of curve crossing as observed in the U-Net network during training. Once both the U-Net network and the improved network reach convergence, the pixel accuracy and average cross-merge ratio values for both networks are presented in Table 6.

**Table 6:** Values of accuracy and average cross-merge ratio of the network before and after improvement.

Models	Training set		Validation set	
	Accuracy	mIoU	Accuracy	mIoU
U-Net	0.9686	0.9181	0.90.38	0.8334
Ours	0.9796	0.9464	0.90.89	0.8667

Based on the data presented in Table 6, our network exhibits higher training and validation accuracies than U-Net. Specifically, our network achieves a 1.10% improvement in training accuracy and a 0.51% improvement in validation accuracy compared to U-Net. Additionally, our network demonstrates higher average cross-merge ratios for both training and validation, with a 2.83% increase for training and a 3.33% increase for validation, under the combined dataset of optical and SAR.

**Figure 10:**
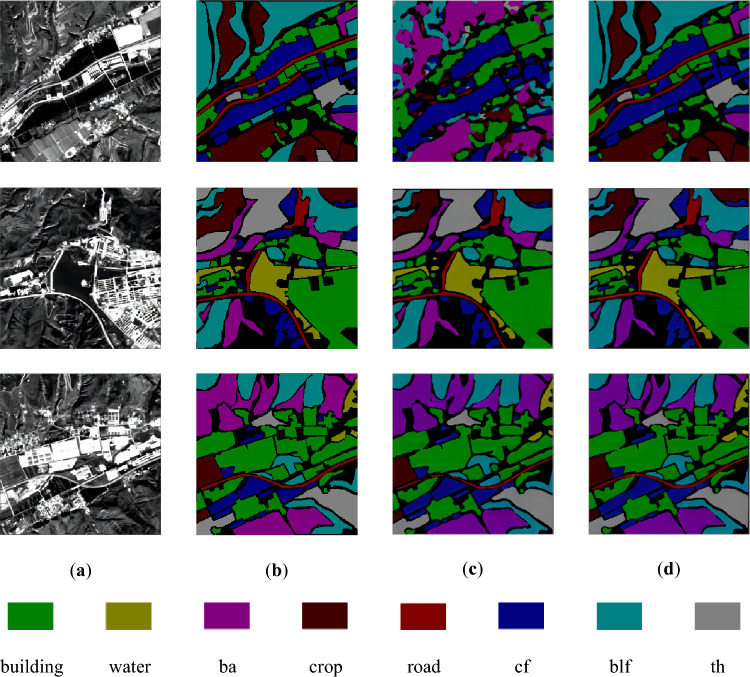
Comparison of network prediction results before and after improvement. (**a**) Original image; (**b**) real labels; (**c**) results of U-Net; (**d**) results of improved network.

A comparison of the prediction results of the U-Net network and the improved network are shown in Fig. 10.

Upon observing (c) in Fig. 10, several issues can be identified in the prediction results of U-Net. Firstly, although roads can be identified, there is a significant discrepancy between the identified range and the actual labels. Secondly, only a few cultivated lands are correctly classified, with most being incorrectly categorized as bare land or broadleaf forest. Thirdly, some broadleaf forests are misclassified as bare land or mixed forests, and some mixed forests are misclassified as broadleaf forests. By comparing the prediction results of the two networks, it becomes evident that the improved network can accurately identify all eight terrains in the combined dataset. Its recognition range and accuracy are more satisfactory compared to U-Net. Additionally, the improved network demonstrates a higher classification accuracy for cropland, avoiding misclassifications as bare land. Moreover, the improved network exhibits better performance in terms of road classification range and accuracy compared to U-Net.

By examining the data in Table 7, several conclusions can be drawn: (1) the classification accuracy and average intersection ratio of both networks are higher on the combined dataset compared to the individual datasets. This finding confirms that the combined images effectively leverage the spectral terrains of optical images and the texture and plan terrains of SAR images. (2) The improved network achieves validation accuracies of 90.89%, 86.09%, and 89.05% for the combined dataset, Sentinel-1 dataset, and Sentinel-2 dataset, respectively. These values are 0.51%, 2.41%, and 1.84% higher than those of U-Net.

**Table 7:** Values of accuracy and average cross-merge ratio of the network before and after improvement for the three datasets.

Models	Types of datasets	Training set		Validation set	
U-Net	Sentinel-2	Train_acc	0.9477	Valid_acc	0.8721
		Train_miou	0.8348	Valid_miou	0.7411
	Sentinel-1	Train_acc	0.9606	Valid_acc	0.8368
		Train_miou	0.8894	Valid_miou	0.7566
	Combination	Train_acc	0.9686	Valid_acc	0.9038
		Train_miou	0.9181	Valid_miou	0.8334
FCN	Sentinel-2	Train_acc	0.8371	Valid_acc	0.7645
		Train_miou	0.6690	Valid_miou	0.5838
	Sentinel-1	Train_acc	0.8612	Valid_acc	0.8368
		Train_miou	0.7537	Valid_miou	0.5930
	Combination	Train_acc	0.9405	Valid_acc	0.8664
		Train_miou	0.6863	Valid_miou	0.6461
DeepLabV3	Sentinel-2	Train_acc	0.7825	Valid_acc	0.7396
		Train_miou	0.6273	Valid_miou	0.5594
	Sentinel-1	Train_acc	0.8418	Valid_acc	0.7453
		Train_miou	0.6230	Valid_miou	0.5554
	Combination	Train_acc	0.8858	Valid_acc	0.8020
		Train_miou	0.6623	Valid_miou	0.5928
Ours	Sentinel-2	Train_acc	0.9635	Valid_acc	0.8905
		Train_miou	0.9159	Valid_miou	0.8104
	Sentinel-1	Train_acc	0.9741	Valid_acc	0.8609
		Train_miou	0.9408	Valid_miou	0.7874
	Combination	Train_acc	0.9796	Valid_acc	0.9089
		Train_miou	0.9464	Valid_miou	0.8667

### Ablation experiments

The conventional down-sampling method of employing a pooling layer has been modified to implement a convolutional approach with a step size of 2. The CBAM has been integrated into the U-Net network's terrain extraction architecture. Additionally, the ReLU function, originally utilized in the U-Net, has been substituted with the Leaky ReLU function as the activation function. To assess the effectiveness of these modifications to the network, four distinct schemes were devised.

Scheme I is grounded on the traditional U-Net network, incorporating the CBAM attention mechanism solely in the terrain extraction phase.

Scheme II builds upon Scheme I by designating the Leaky ReLU function as the network's activation function.

Scheme III evolves from Scheme I by adopting a convolution with a step size of 2 to replace the pooling layer for down-sampling.

Lastly, Scheme IV, developed from Scheme III, employs the Leaky ReLU function as the network's activation function.

The experiment's initial learning rate was established at 0.0001, and the network underwent 200 iteration rounds. Table 8 presents the experimental findings using the Sentinel-2 optical dataset, Table 9 displays the results from the Sentinel-1 SAR dataset, and Table 10 outlines the findings from the combined dataset.

**Table 8:** Experimental results of Sentinel-2 optical dataset.

Method	Train_acc	Train_miou	Valid_acc	Valid_miou
U-Net	0.9477	0.8348	0.8721	0.7411
Scheme I	0.9539	0.8611	0.8794	0.7673
Scheme II	0.9571	0.8736	0.8815	0.7806
Scheme III	0.9628	0.8993	0.8879	0.8033
Scheme IV	0.9635	0.9159	0.8905	0.8104

**Table 9:** Experimental results of Sentinel-1 optical dataset.

Method	Train_acc	Train_miou	Valid_acc	Valid_miou
U-Net	0.9606	0.88.94	0.8368	0.7566
Scheme I	0.9649	0.90.27	0.8440	0.7695
Scheme II	0.9662	0.91.55	0.8471	0.7819
Scheme III	0.9685	0.93.38	0.8586	0.7852
Scheme IV	0.9741	0.94.08	0.8609	0.7874

**Table 10:** Experimental results of the combined dataset.

Method	Train_acc	Train_miou	Valid_acc	Valid_miou
U-Net	0.9686	0.9181	0.9038	0.8334
Scheme I	0.9712	0.9278	0.9055	0.8399
Scheme II	0.9724	0.9315	0.9064	0.8436
Scheme III	0.9777	0.9390	0.9082	0.8573
Scheme IV	0.9796	0.9463	0.9089	0.8667

An examination of the data presented in the aforementioned three tables reveals that Scheme IV outperforms the others in terms of classification accuracy and mIoU values. The empirical results suggest that the inclusion of the CBAM attention mechanism during the terrain extraction stage, the application of a convolution with a stride of 2 for down-sampling in place of pooling, and the implementation of the Leaky ReLU function as the activation function contribute to an appreciable improvement in network classification accuracy, albeit not substantially. The empirical evidence indicates that the synergistic implementation of the CBAM attention mechanism, convolution in lieu of pooling, and Leaky ReLU function results in a more substantial enhancement of network performance.

## Conclusions

This paper proposes an enhanced U-Net network that incorporates a convolutional block attention mechanism, an alternative activation function, and a convolutional substitution pool. Firstly, three distinct datasets are created and subjected to data augmentation techniques to increase their size. Subsequently, the terrain extraction stage incorporates the convolutional block attention mechanism to enhance the network's attention to terrains in two dimensions. Secondly, the Leaky ReLU function replaces the ReLU function in the original U-Net to prevent gradient death-induced weight issues during network training. Lastly, down-sampling is achieved through a convolution operation with a step size of 2, rather than using Max pooling. By using convolution instead of pooling and by changing the original convolution kernel size and number, the number of parameters and floating-point computations of the network are effectively reduced. Experimental results demonstrate that our proposed network surpasses the performance of the U-Net in the classification of all three datasets, showing improvements in PA, mIoU, and the number of parameters. These results validate the effectiveness of the convolutional block attention mechanism and the substitution of pooling operations with convolution in the field of land cover classification. However, the band combination does not fully exploit the advantages of image fusion, although it can improve the resolution of the image and realize a certain degree of information complementarity. Therefore, future research directions for this study encompass the following aspects: (1) fusion of high-resolution remote sensing images at both the pixel and terrain layers to maximize the utilization of image terrains and enhance classification accuracy; (2) exploration of additional high-resolution remote sensing images and diverse terrain types for further experimentation and investigation; and (3) investigation of land cover classification algorithms in terms of network depth and operational efficiency, aiming to enhance the classification accuracy and applicability of the network through continuous improvement of the network model.

## Data Availability

All data has been included in study.
